# A Novel Insight into Endothelial and Cardiac Cells Phenotype in Systemic Sclerosis Using Patient-Derived Induced Pluripotent Stem Cell

**DOI:** 10.22074/cellj.2021.7244

**Published:** 2021-07-17

**Authors:** Sedigheh Gholami, Zahra Mazidi, Sara Pahlavan, Fariba Moslem, Mahya Hosseini, Adeleh Taei, Mahdi Hesaraki, Maryam Barekat, Nasser Aghdami, Hossein Baharvand

**Affiliations:** 1. Department of Stem Cells and Developmental Biology, Cell Science Research Center, Royan Institute for Stem Cell Biology and Technology, ACECR, Tehran, Iran; 2. Department of Developmental Biology, University of Science and Culture, Tehran, Iran; 3. Department of Regenerative Medicine, Cell Science Research Center, Royan Institute for Stem Cell Biology and Technology, ACECR, Tehran, Iran

**Keywords:** Angiogenesis, Cardiomyocyte, Induced Pluripotent Stem Cells, Systemic Sclerosis, VE-Cadherin

## Abstract

**Objective:**

Systemic sclerosis (SSc) is a connective tissue disease associated with vascular damage and multi organ fibrotic changes with unknown pathogenesis. Most SSc patients suffer from defective angiogenesis/vasculogenesis and cardiac conditions leading to high mortality rates. We aimed to investigate the cardiovascular phenotype of SSc by cardiogenic differentiation of SSc induced pluripotent stem cells (iPSC).

**Materials and Methods:**

In this experimental study, we generated iPSC from two diffuse SSc patients, followed by successful differentiation into endothelial cells (ECs) and cardiomyocytes (CMs).

**Results:**

SSc-derived EC (SSc-EC) expressed KDR, a nearly EC marker, similar to healthy control-EC (C1-EC). After sorting and culturing KDR+ cells, the resulting EC expressed CD31, a late endothelial marker, but vascular endothelial (VE)-cadherin expression markedly dropped resulting in a functional defect as reflected in tube formation failure of SSc-EC. Interestingly, upregulation of SNAI1 (snail family transcriptional repressor 1) was observed in SSc-EC which might underlie VE-cadherin downregulation. Furthermore, SSc-derived CM (SSc-CM) successfully expressed cardiac- specific markers including ion channels, resulting in normal physiological behavior and responsiveness to cardioactive drugs.

**Conclusion:**

This study provides an insight into impaired angiogenesis observed in SSc patients by evaluating in vitro cardiovascular differentiation of SSc iPSC.

## Introduction

Systemic sclerosis (SSc) develops as a chronic connective tissue disease which involves multiple organs; however, its etiology remains unknown. It is characterized by vascular injury, immune dysregulation and extensive fibrosis of several organs including the skin (1). The complex pathogenesis of SSc remains unclear, however it is known that genetic, epigenetic and environmental factors contribute to its development (2). Endothelial cell (EC) injury is one of the first phases in pathogenesis of SSc. The damaged endothelium upregulates the expression of adhesion molecules and chemokines resulting in recruitment of inflammatory cells. Multiple cytokines and growth factors, secreted by inflammatory and immune cells, promote activation and differentiation of resident fibroblasts into myofibroblasts, which cause excessive extracellular matrix (ECM) proteins production leading to fibrosis. Thus, suggested pathogenesis of SSc includes a complex interplay between vascular abnormality, inflammation and autoimmunity, as well as fibrosis (3).

Clinical and *in vitro* studies demonstrated an impaired angiogenesis in SSc. Moreover, several studies suggested that ECs might be the origin of a subset of activated fibroblasts or myofibroblasts. Furthermore, endothelial-mesenchymal transition (EndoMT) and their differentiation into collagen-producing cells are likely to represent an additional source of extra collagen (4).

In addition to vascular complications, many cases of heart phenotypes have been reported in SSc which accounts for 11-36% mortality in these patients (5). Cardiac manifestation of SSc can be caused directly 

by a myocardial involvement developing to myocardial fibrosis or indirectly by pulmonary arterial hypertension or systemic hypertension as the possible outcome of pulmonary and renal involvements (6). Not only the heart wall including epicardium, myocardium *and *endocardium, but also coronary arteries, cardiac valves and nervous system may be affected in SSc leading to heart failure (7). A meta-analysis done by Komócsi et al. (8), confirmed that cardiopulmonary manifestation is the main cause of mortality in SSc patients. Despite cohort studies suggesting a decline in mortality risk of SSc, a meta-analysis of cohort studies, conducted by Elhai et al. (9) reported no substantial changes in standardized mortality ratio (SMR) over 40 years. 

Over the last decade, molecular studies-based clinical trials provided more knowledge on the pathogenesis of scleroderma (10), however, further investigations are still needed. Such molecular studies require appropriate animal or cell-based models recapitulating scleroderma phenotype. Patient-specific induced pluripotent stem cells (iPSCs) allow us to examine the disease phenotype in target tissue as well as other cell types of body (11). In the present study, we produced iPSC from skin biopsies of two SSc patients and characterized them followed by cardiogenic differentiation into cardiomyocytes (CMs) and ECs and their characterization.

## Materials and Methods

### Generation of patient-specific induced pluripotent stem cells

**Fig.1:**
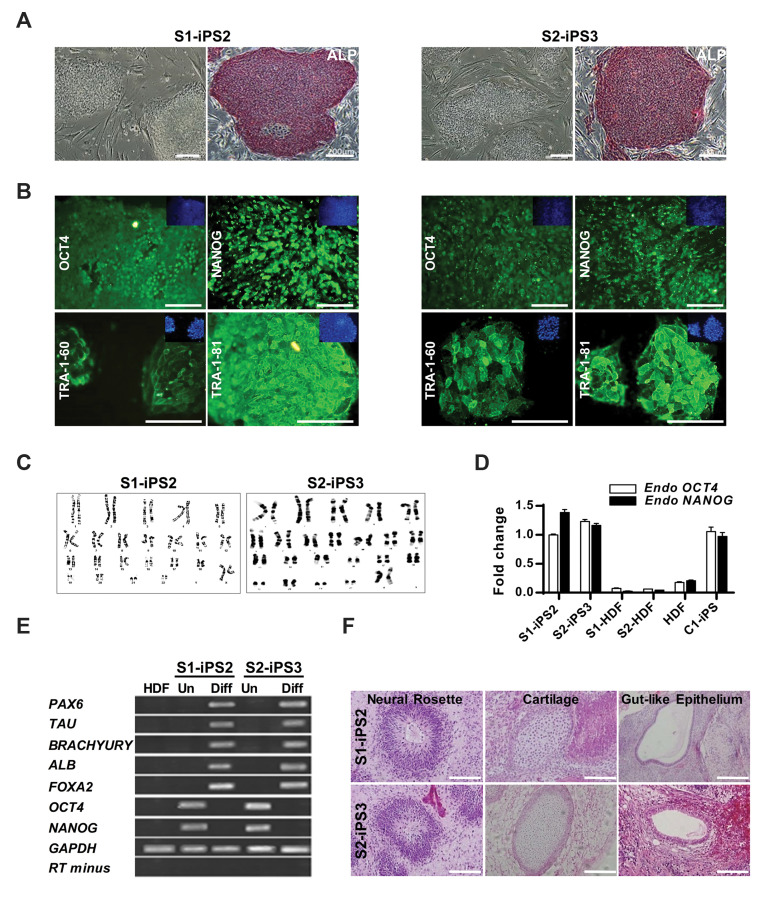
Characterization of established SSc iPSC. **A.** Phase contrast microscopy of established SSc iPSC clones [S1-iPS2 (patient 1) and S2-iPS3 (patient 2)] and ALP staining of derived iPSC (scale bar: 200 µm), **B.** Immunofluorescence staining demonstrated the expression of pluripotency markers (OCT4, NANOG, TRA-1-60 and TRA-1-81) in derived iPSC. Nuclei were counterstained with DAPI (scale bar: 100 µm), **C. **Both S1-iPS2 and S2-iPS3 lines maintained normal karyotype, **D.** qRT-PCR analysis showed endogenous *OCT4* and *NANOG* expression in SSc iPSC to levels similar to those of healthy control-iPSC (C1-iPSC) while were silent in the HDF. Fold change was calculated by ∆∆Ct method and expression of each gene was normalized against *GAPDH*, **E.** RT-PCR analyses indicated the expression of differentiation markers for the three germ layers by EB-mediated differentiation (Diff) in comparison with undifferentiated state (Un), and **F.** Tissue morphology of teratoma derived from SSc iPSC (scale bar: 100 µm). Data are represented as mean ± SEM, n=3 (biological replicate). SSc; Systemic sclerosis, iPSC; Induced pluripotent stem cells, ALP; Alkaline phosphatase, qRT-PCR; Quantitative real-time polymerase chain reaction, HDF; Human dermal fibroblasts, and EB; Embryoid body.

In this experimental study, human dermal fibroblasts of SSc patients were digested using 0.1% collagenase I (Sigma, USA) and cultured in fibroblast medium (Dulbecco’s modified Eagle’s medium [DMEM, Gibco, USA]) enriched with 10% fetal bovine serum (FBS, Gibco, USA) and 1% penicillin and streptomycin (Gibco, USA), as previously described (12). Institutional review board approval by Royan Institute Ethics Committee’s general principles in compliance with the declaration of Helsinki (IR ACECR, ROYAN REC, 1395 175) and consent from patients for iPSC derivation, were obtained. The clinical features of the SSc patients are shown in Table S1 (See Supplementary Online Information at www. celljournal.org). To generate patient-specific iPSC, dermal fibroblasts were reprogrammed by four Yamanaka factors (*OCT4, SOX2, KLF4,* and *c-MYC*) delivered by retrovirus in serum- and feeder-free conditions based on a previously reported protocol (12). On transduction day 6, fibroblast medium was exchanged with human embryonic stem cell (hESC) medium supplemented with 100 ng/ml basic fibroblast growth factor (bFGF, Royan Biotech, Iran). hESC medium was *comprised of* DMEM/F12 (Gibco, USA) supplemented with 20% Knockout serum replacement (KOSR, Gibco, USA), 1% non-essential amino acids (Gibco, USA), 1% penicillin and streptomycin, 2 mM L-glutamine (Gibco, USA) and 0.1 mM β-mercaptoethanol (Sigma, USA). At day 14-20 of re-plating, embryonic stem cells (ES)- like colonies were observed (Fig.1A). For each patient, three iPSC clones were established for subsequent analyses. Due to same characteristics, we followed our experiment on one clone for each patient. Two control iPSC lines, one derived from a healthy Iranian 40-year-old male (iPSC4 abbreviated as C1) (12) and the other obtained from a healthy Iranian 32-year-old female (B-iPSC11 abbreviated as C2) (13) were acquired from the Stem Cell Bank of Royan Institute. Following thawing, iPSC was cultured on mitomycin C-treated mouse embryonic fibroblast (MEF) feeder cells in hESC medium supplemented with 5 ng/ml bFGF. For expansion, iPSCs were passaged on ECM Gel (Sigma, USA, 1:30) using collagenase IV (0.5 mg/ml, Gibco, USA): dispase (1 mg/ml, Gibco, USA) at the ratio of 1:2. 

### Karyotype analysis

Karyotyping was performed at cytogenetic laboratory of the Institute for Human Genetics (Royan Institute, Iran) according to a standard procedure described previously (14). Briefly, 70% confluent human iPSC (hiPSC) colonies were treated with 0.66 µM thymidine (Sigma-Aldrich, USA) at 37˚C overnight. Then, cells were washed and rested for 5 hours before being exposed to colcemid (Gibco, USA, 0.15 µg/ml) for 30 minutes. Afterwards, trypsinized cells were treated with 0.075 M KCl and fixed. Karyotyping was performed using standard G-band staining. 

### *In vitro* spontaneous differentiation of human induced pluripotent stem cells

To evaluate the spontaneous differentiation capacity of the above-noted derived hiPSC lines into three embryonic germ layers, we generated embryoid body (EB). Briefly, hiPSC colonies were dispersed into single cells using Accutase (Sigma, USA) and transferred into non-adhesive bacterial plates (Greiner Bio-One, Germany) containing DMEM/F12 medium supplemented with 20% KOSR, 1% non-essential amino acids, 2 mM L-glutamine, and 0.1 mM β-mercaptoethanol without bFGF. After 12 days, the generated EBs were plated on ECM Gel-coated culture dishes for another 8 days. On day 21 of spontaneous differentiation, EB samples were collected and ectoderm, mesoderm and endoderm differentiation were evaluated with respect to transcriptional expression of each germ layer’s specific genes using quantitative real-time polymerase chain reaction (qRT-PCR).

### Teratoma formation

Sc iPSC (2×10^6^) were suspended in phosphate-buffered saline (PBS) and injected into the subrenal capsule of 8-week-old NOD/SCID mice (BioLASCO) using a 26-gauge syringe (BD Biosciences, USA). Eight weeks after injection, tumors were harvested, weighed and fixed in 10% formalin. After fixation, tumors underwent histological analyses for ectoderm, mesoderm and endoderm formation.

### Immunofluorescence and alkaline phosphatase staining

Cells were fixed in 4% paraformaldehyde at room temperature (RT) for 25 minutes, washed with PBS/0.05% Tween 20 and permeabilized using 0.5% Triton X-100 in PBS for 30 minutes at RT. Thereafter, cells were washed and blocked in blocking solution (1% bovine serum albumin [BSA, Life Technology] in PBS) for 1 hour at RT. Primary antibodies were diluted in blocking solution and added to the cells overnight at 4˚C. Cells were then washed three times with PBS/0.05% Tween 20, each time for 5 minutes, and incubated with appropriate secondary antibodies in blocking solution for 45 minutes at RT. Lastly, cells were washed three times with PBS/0.05% Tween 20 and nuclei were counterstained with 4’,6-diamino-2- phenylindole (DAPI, Sigma, USA). Images were captured using a fluorescent microscope (IX71, Olympus, Japan). Antibodies used in the present study are mentioned in Table S2 (See Supplementary Online Information at www.celljournal.org).

We performed Alkaline phosphatase (ALP) staining based on the manufacturer’s instructions (Sigma, USA).

### Cardiac and endothelial differentiation of human induced pluripotent stem cells

hiPSC colonies were dispersed into single cells by 4-5 minutes treatment with Accutase (Sigma-Aldrich, USA) at 37˚C and incubated in non-adhesive bacterial plates (Greiner Bio-One, Germany) at the density of 2×10^5^ cells/ml in hESC medium. After aggregate formation, the directed cardiogenic differentiation of hiPSC was performed in static suspension culture using a cocktail of small molecules (SM) as previously described (15). Briefly, hiPSC aggregates with average diameter of 175 ± 25 μm were treated with 12 μM of SM CHIR99021 (CHIR, Stemgent, USA) in differentiation medium (RPMI 1640 (Gibco, USA) supplemented with 2% B27 without retinoic acid (Gibco, USA), 2 mM L-glutamine (Gibco, USA), 0.1 mM β-mercaptoethanol (Sigma, USA), 1% nonessential amino acids (Gibco, USA), 1% penicillin and streptomycin). After 24 hours, aggregates were washed with Dulbecco’s PBS (DPBS, Gibco, USA) and maintained in fresh differentiation medium for 24 hours. On differentiation day 2, the medium was changed with new differentiation medium containing 5 μM IWP2 (Tocris Bioscience, UK), 5 μM SB431542 (Sigma-Aldrich, USA) and 5 μM purmorphamine (Pur, Stemgent, USA) for 48 hours. On day 4, the aggregates were washed with DPBS and cultured in fresh differentiation medium which was refreshed every 2 or 3 days. In order to determine the efficiency of cardiac differentiation, we counted the number of beating spheroids on a daily basis, starting with the first beating observation, using an inverted cell culture microscope (Olympus, Japan). For CM immunostaining, the 30-day-post-differentiation beating spheroids were subjected to enzymatic digestion by 4-5 min treatment with Accutase at 37˚C followed by gentle pipetting and plated at the density of 8×10^4^ cell/ cm^2^ on ECM-Gel (Sigma, USA, 1:30) coated 4-well tissue-culture plate in differentiation medium. After adequate cell attachment, CM were fixed and all steps of immunofluorescence staining were taken as described in supplementary material.

For endothelial differentiation, we used a previously reported protocol with some modifications (16). hiPSC aggregates with average diameter of 200-250 µm were treated with 12 µM of CHIR99021 in RPMI medium supplemented with B27 without retinoic acid for 24 hours. Cells were then incubated in RPMI/B27 without SM for another 24 hours. On day 2 of differentiation, cells were treated with 25 ng/ml BMP4 (R&D), 10 µM Purmorphamine, 10 µM SB431542 and 50 ng/ml VEGF-A (Royan-Biotech, Iran) in RPMI/B27 medium for 48 hours. Next, the medium was exchanged with EGM-2 (Lonza, Switzerland) supplemented with 50 ng/ ml VEGF-A and cells were incubated for another 48 hours. On day 6, differentiated aggregates were dispersed into singles cells using Accutase and sorted based on KDR expression (R&D, USA). KDR^+^ sorted cells were sub-cultured on collagen type I-coated plates (10 μg/cm^2^, Sigma-Aldrich, USA) in EGM-2 medium containing 50 ng/ml VEGF-A until reaching appropriate confluency

### Gene expression analyses

Total RNA was extracted using TRIzol reagent (Sigma-Aldrich, USA). Toprevent DNA contamination, extracted RNA was treated with RNase-free DNase I (Takara, Japan). cDNA synthesis was performed using a PrimeScript^TM^ RT Reagent Kit (Perfect Real Time) (Takara, Japan) based on the manufacturer’s instructions and qRT-PCR was performed using a SYBR Premix Ex Taq Kit (Takara Bio. Inc, Japan) and a Rotor Gene Corbett System (Corbett Life Science, Australia). Results were analyzed by Rotor-Gene 6000 analysis software (Corbett Life Science, Australia, version 1.7). All experiments were done in triplicate. The relative gene expression level of the desired genes was calculated by ∆∆CT method and normalized against the housekeeping gene, glyceraldehyde 3-phosphate dehydrogenase (*GAPDH*). All primer sequences used in the present work, are listed in Table S3 (See Supplementary Online Information at www.celljournal.org).

### Flow cytometry and cell sorting

Differentiated hiPSC aggregates were collected at particular time-points of differentiation and dissociated into single cells using Accutase solution and 0.05% trypsin/ ethylenediaminetetracetic acid (Gibco, USA) for hiPSC-CM and hiPSC-EC, respectively. Then, single cells were fixed by treatment with 4% paraformaldehyde for 20 minutes at 4˚C. After washing with PBS/0.05% Tween 20, the fixed cells were permeabilized by 0.2% Triton X-100 in PBS for 30 minutes at RT, blocked in serum and stained, either 1 hour for surface markers or overnight for cytoplasmic markers, with appropriate primary antibodies at 4˚C. Cells were then washed and incubated with appropriate secondary antibody for 1 hour at RT. Cells were analyzed using a BD FACS Calibur flow cytometer (BD Biosciences, USA). Data analysis was done by Flowing Software (version 2.5.1, Turku Centre for Biotechnology, Finland).

For sorting, cells were dissociated into single cells using 0.05% trypsin/EDTA on day 6 of endothelial differentiation. Cells were then washed in PBS containing 2% FBS (FACS buffer) and incubated with anti-human KDR for 1 hour at 4˚C. After washing, KDR-positive cells were sorted using FACS Calibur.

### Uptake of acetylated low-density lipoprotein (Dil-ac-LDL

ECs were incubated with 10 µg/ml of acetylated low-density lipoprotein (Dil-Ac-LDL, Biomedical Technology, UK) for 4 hours at 37˚C. Then, cells were fixed by treatment with 4% paraformaldehyde for 10 minutes at RT. After washing with PBS, nuclei were counterstained with DAPI (DAPI, Sigma, USA) and visualized using a fluorescence microscopy (IX71; Olympus, Japan).

### Tube formation assay

ECMatrix™ (In Vitro Angiogenesis Assay Kit, Chemicon, USA) was aliquoted into all wells of a 96-well plate (50 µl/well) and incubated for 1-2 hour at 37˚C to polymerize. Thereafter, 10^4^ cells/well were seeded onto the matrix in 150 μl of EGM-2 medium and incubated for 2 hours at 37˚C with 5% CO_2_ . Tube formation was assessed using Olympus CKX41 inverted microscope and analyzed using “Image J” software.

### Multielectrode array recording

A multielectrode array (MEA) data acquisition system (Multi Channel Systems, Reutlingen, Germany) was used to record the extracellular field potential (FP) of hiPSC-CM. The MEA plate is composed of 60 titanium nitride electrodes with an inter-electrode space of 200 µm. Beating spheroids were plated on ECM Gel-coated MEA plates and allowed to attach for 48-72 hours before recording. Baseline FP recording and drug testing were performed 30 ± 5 days post-differentiation. On the day of the experiment, the MEA plates were connected to a head stage amplifier. FP was acquired at 2 kHz, and all recordings were performed at 37˚C. Signals were recorded for 60 seconds at baseline and 5 minutes after drug application. All drugs were purchased from Sigma-Aldrich, otherwise stated. Stock solutions were prepared daily in appropriate solvent and used at desired concentrations made in RPMI/B27 medium. Data were analyzed by Cardio2D software (version 2.2.2.0, Multichannel system MCS GmbH). FP durations (FPD) was normalized to beating rate using the Bazett correction formula (corrected FPD [cFPD]=FPD/^3^ √ (RR interval)). Drugs used in pharmacological studies are listed in Table S4 (See Supplementary Online Information at www.celljournal.org).

### Whole cell patch-clamp recording

Action potential (AP) was recorded from spontaneously beating hiPSC-CM using the current-clamp mode of the whole cell patch-clamp configuration. On differentiation day 30, beating spheroids were dissociated into single cells by Accutase accompanied by gentle pipetting. Single beating CM were plated onto ECM-Gel coated glass coverslips and incubated at 37˚C overnight. Then, the cover slips were transferred to a recording chamber mounted on the stage of an Olympus inverted microscope (Olympus, Japan). The bath solution within the chamber contained 135 mM NaCl, 5.4 mM KCl, 10 mM HEPES, 10 mM D-glucose, 1 mM MgCl, and 1.8 mM CaCl_2_ and the pH was adjusted to 7.4 using NaOH. Recording pipettes were pulled from borosilicate glass capillaries (Harvard Apparatus, Holliston, MD) by a P-97 horizontal puller (Sutter Instrument, Novato) to a tip resistance of 3-6 MΩ. The pipette solution contained 135 mM KCl, 10 mM NaCl, 1 mM EGTA, 10 mM HEPES, and 5 mM MgATP, and the pH was adjusted to 7.2 using KOH.

Data were acquired using a multiclamp 700B amplifier (Axon Instruments, Molecular Devices Corp., Union City, CA, USA), a Digidata 1440 analog-to-digital board and pClamp 10 software (Axon Instruments), at a sampling frequency of 10 kHz and low-pass filtered at 2 kHz. Data analysis was performed using Clampfit 10 (Axon Instruments) and Prism 6 (GraphPad Software, La Jolla, CA, USA) software.

### Ca^+2^ imaging

In order to record Ca^+2^ transients in hiPSC-CM, beating spheroids were incubated with 1 µM Fura-2 AM (Sigma-Aldrich, USA) for 30 minutes at 37˚C, 30-day post-differentiation. Then, beating spheroids were washed with RPMI/B27 medium for 15 minutes at 37˚C. Afterwards, Ca^+2^ imaging was performed using a fluorescent microscope (IX71, Olympus, Japan) equipped with a DP72 digital camera (Olympus, Japan) and analyzed in a custom- made Matlab macro. Calcium transient amplitude and calcium transient duration at 80% decay (CTD80) were calculated. In order to study the Ca^+2^ content of sarcoplasmic reticulum (SR), rapid puffs of 10 mM caffeine were applied and Ca^+2^ release was imaged. Fractional Ca^+2^ release (FCR) from the stores was calculated as the ratio of the amplitude of Ca^+2^ transient to the amplitude of the caffeine-induced Ca^+2^ release. 

### Statistical methods

All data are presented as mean ± SEM from at least three independent biological replicates for each hiPSC line. Comparisons were made by analysis of variance (ANOVA, one-way and two way) or unpaired t test when appropriate, using GraphPad Prism version 6.01 (GraphPad Software, La Jolla California, USA) and considered significant when P<0.05.

### 

The datasets generated during and/or analysed during the current study are available from the corresponding author on reasonable request.

## Results

### Systemic sclerosis fibroblasts were successfully reprogrammed to pluripotency *in vitro*

To generate SSc iPSC, primary fibroblasts were obtained from skin biopsy of each patient following receiving an informed written consent. Cells were reprogrammed with Yamanaka factors using retroviral vectors. After 20 days, hESC-like colonies were picked and expanded for characterization (Fig.1A). Both SSc iPSC lines revealed strong ALP activity (Fig.1A) and expressed major hESC-specific markers (OCT4, NANOG, TRA-1-60, and TRA-1-81, Fig.1B). Furthermore, a normal karyotype was determined for both SSciPSC lines (S1- iPS2 and S2-iPS3) indicating chromosomal stability during iPSC generation (Fig.1C). To further confirm reprogramming to pluripotency, the expression of *OCT4* and *NANOG* was evaluated which revealed that both S1-iPS2 and S2-iPS3 expressed endogenous *OCT4* and *NANOG* (Fig.1D). In addition, qRT-PCR analysis showed silencing of exogenous genes (*OCT4, c-MYC, KLF4* and *SOX2*) in derived S1-iPS2 and S2-iPS3 (Fig. S1, See Supplementary Online Information at www. celljournal.org). Differentiation potency of S1-iPS2 and S2-iPS3 into embryonic germ layers was evaluated by EB formation. To achieve that, iPSC were cultured in suspension for 12 days to form EB in the absence of bFGF and then transferred to ECM Gel-coated dishes for another 8 days. RT-PCR analyses demonstrated that ectoderm (*PAX6* and *TAU*), mesoderm (*Brachyury*) and endoderm (*ALB* and *FOXA2*) specific markers were only expressed in differentiated S1-iPS2 and S2-iPS3but not in undifferentiated state (Fig.1E). Furthermore, spontaneous differentiation of iPSC lines resulted in the development of ectoderm, mesoderm and endoderm cells as reflected by upregulation of *SOX1/PAX6, MESP1/Brachyury* and *FOXA2/AFP* genes as specific markers of each germ layer, respectively (Fig.S2, See Supplementary Online Information at www.celljournal.org). Moreover, SSci PSC were transplanted into the subrenal capsule of 5-week-old NOD mice. After about 7 weeks, teratoma was formed, harvested and subjected to immunohistochemistry analyses. Our results demonstrated the presence of cartilage (mesoderm), gut-like epithelium (endoderm) and neural rosette (ectoderm) tissues in teratoma which further confirmed stemness of iPSC (Fig.1F). Altogether, these data showed that SSc fibroblasts were successfully reprogrammed to pluripotency.

### Systemic sclerosis induced pluripotent stem cells were successfully differentiated into endothelial progenitor cells

**Fig.2:**
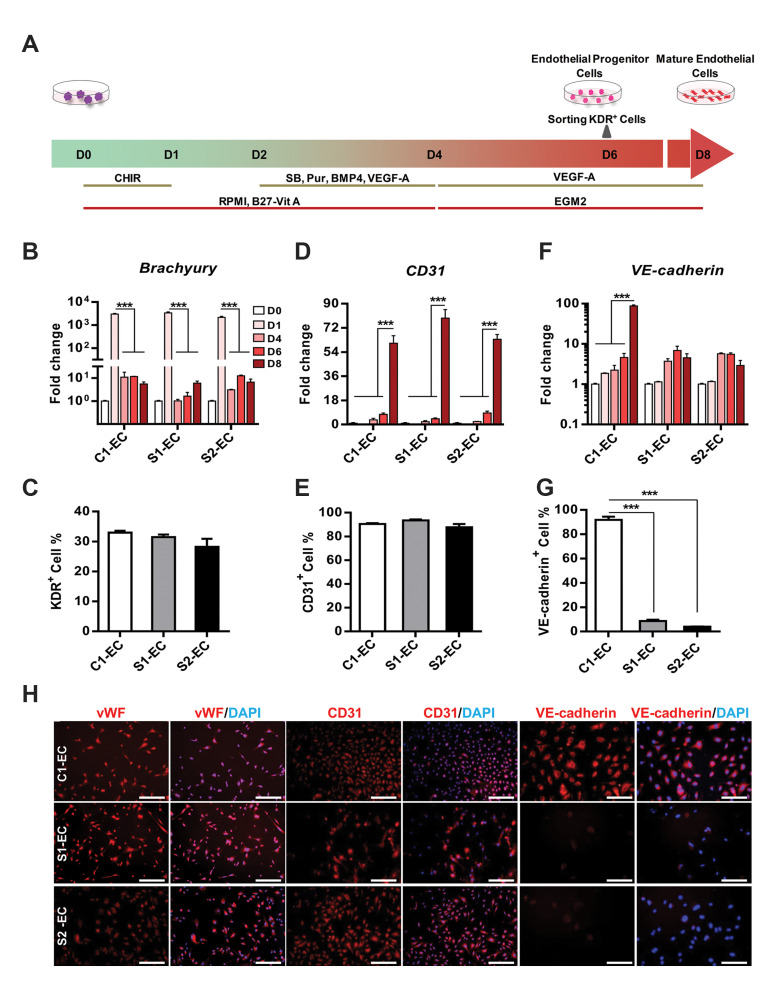
Differentiation of SSc iPSC toward endothelial lineage. **A.** A schematic diagram illustrating the endothelial differentiation protocol in which growth factors and small molecules were used, **B.** The relative expression of *Brachyury* gene during differentiation as assessed by qRT-PCR, **C.** Flow cytometry analysis of KDR expression on differentiation day 6 demonstrated no significant difference between SSc-EC and C1-EC, **D.** Similar expression of *CD31* at mRNA and** E.** At protein levels in iPSC-derived EC, **F.** qRT-PCR analysis demonstrated that *VE-cadherin* was significantly downregulated in SSc-EC compared to C1-EC. The expression of each gene was normalized against *GAPDH*. The relative expression was calculated by ∆∆Ct method (undifferentiated state ‘’D0’’ was set at 1), **G.** Expression level of VE-cadherin protein was measured by flow cytometry, and **H. **Immunofluorescence staining demonstrated the expression of vWF, CD31 and VE-cadherin in iPSC-derived EC. Nuclei were counterstained with DAPI (scale bar: 100 µm). All data are represented as mean ± SEM. Comparisons were made by one-way and two-way analysis of variance (***; P<0.001). SSc; Systemic sclerosis, iPSC; Induced pluripotent stem cells, qRT-PCR; Quantitative real-time polymerase chain reaction, VE; Vascular endothelial, EC; Endothelial cells, C1-EC; Healthy control iPSC-EC, S1-EC; SSc1 iPS2-EC, and S2-EC; SSc2 iPS3- EC. n≥3 (biological replicate) for all experiments.

hiPSC lines were differentiated to ECs by a cocktail of growth factors and SMs (Fig.2A). Cells were collected on days 0, 1, 4, 6 and 8 of differentiation in order to evaluate the expression of endothelial genes and proteins. The highest level of *Brachyury* transcriptional expression was observed on day 1 (Fig.2B). The expression of *KDR* was substantially increased on day 6 of differentiation in iPSC lines (40.3 ± 7, 26.4 ± 8 and 15.9 ± 0.6 fold increase compared to undifferentiated state in C1-EC, S1-EC and S2-EC, respectively, Fig.S3A, See Supplementary Online Information at www.celljournal.org). Flow cytometry analyses indicated that almost 30% of cells were KDR-positive (Fig.2C) implicating their identical potential for endothelial progenitor cells (EPC) generation. Although the percentage of KDR expressing cells was similar in C1-EC and both SSc iPSC lines-derived EC, the levels of expression were reduced in SSc-ECs (Fig.S3B, See Supplementary Online Information at www.celljournal. org).

### Systemic sclerosis induced pluripotent stem cells-derived endothelial cells showed defective angiogene

**Fig.3:**
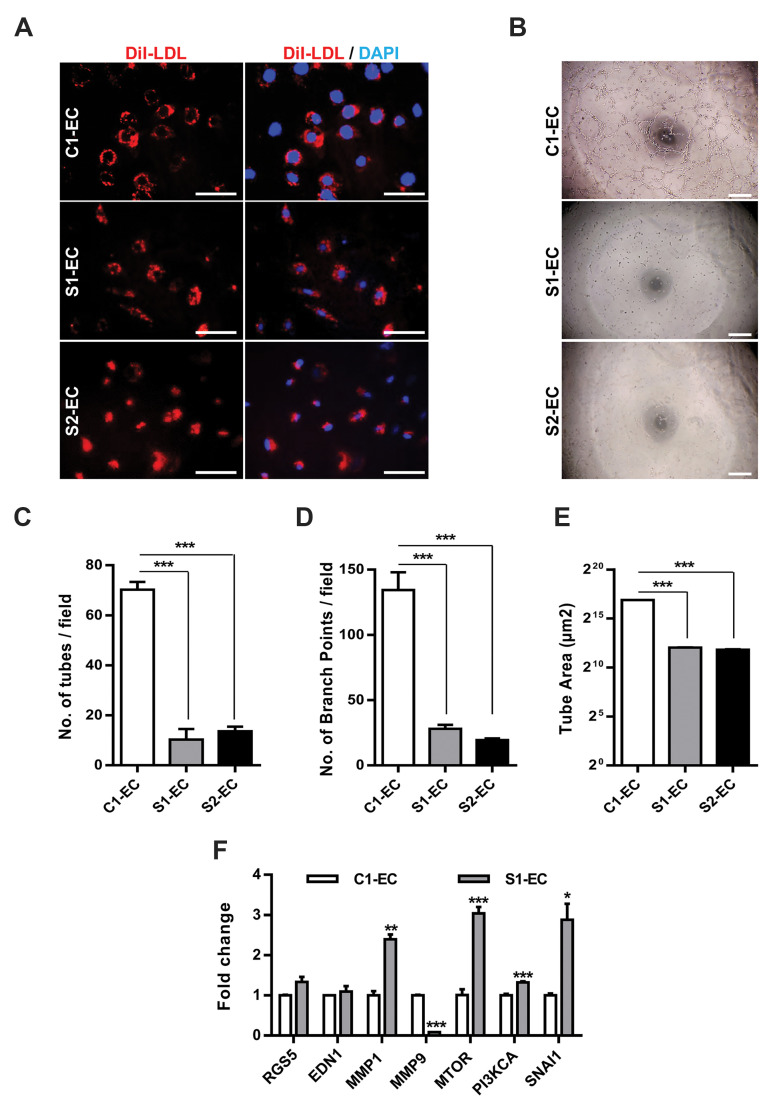
Functional analyses of SSc-EC. **A.** Immunofluorescence staining of C1-EC, S1-EC and S2-EC demonstrated DiI-LDL uptake by iPSC-derived EC. Nuclei were counterstained with DAPI (blue) (scale bar: 50 µm), **B.** SSc-EC did not form tube-like structures *in vitro* (scale bar: 500 µm), **C-E.** Analyses of tube-like structures demonstrated that SSc-EC lacked angiogenic properties, and **F.** Gene expression analysis in iPSC-derived EC. All data are represented as mean ± SEM. Comparisons were made by one-way analysis of variance or unpaired t test when appropriate (* ; P<0.05, * * ; P<0.01, and ***; P<0.001). SSc; Systemic sclerosis, EC; Endothelial cells, iPSC; Induced pluripotent stem cells, C1-EC; Healthy control iPSC-EC, S1-EC; SSc1 iPS2-EC, and S2-EC; SSc2 iPS3-EC. n≥3 (biological replicate) for all experiments.

KDR-positive cells were sorted on day 6 and cultured in VEGF-A supplemented media on collagen I-coated plates to confluency (Fig.2A). Expression of *CD31* (*PECAM-1*) as a late endothelial marker was substantially upregulated on differentiation day 8 (60.5 ± 10, 79.2 ± 11 and 63.4 ± 6 fold increase compared to undifferentiated state in C1- EC, S1-EC and S2-EC, respectively) which was followed by a fairly similar protein expression pattern (90.5 ± 1%, 93.5 ± 1% and 87.7 ± 4% in C1-EC, S1-EC and S2-EC, respectively) (Fig.2D, E). Relative expression of *CD31* on differentiation day 8 was similar in iPSC lines-derived EC (Fig.S3B, See Supplementary Online Information at www.celljournal.org). In contrast, while *VE-cadherin* (*CD144*) expression in C1-EC peaked on differentiation day 8, neither S1-EC nor S2-EC showed upregulation of this endothelial specific marker (Fig.2F, G). C1-EC exhibited 87-fold rise in *VE-cadherin* expression during differentiation and 91.7% of C1-EC were positive for VE-cadherin. However, no more than 8.6 and 3.9% of differentiated S1-iPS2 and S2-iPS3 were CD144^+^ , respectively. Immunostaining for CD31 and CD144 showed a similar pattern to that for gene expression and further confirmed the protein expression results obtained from flow cytometry analyses. Furthermore, ECs derived from both C1-and SSciPSC, expressed endothelial marker vWF (von Willebrand factor) in a similar manner (Fig.2H). To evaluate the functional characteristics of differentiated ECs, the ability to uptake acetylated low-density lipoprotein (AcLDL) as well as tube formation potential was examined. Both SSc iPSC-derived EC (SSc-EC) revealed strong AcLDL uptake capacity when incubated with these lipoprotein particles (Fig.3A). However, these ECs failed to form tubes (Fig.3B). While C1-EC developed vessel-like tubes with around 70 ± 5 tubes/field, 134.3 branch points/field and mean tube area of 0.12 mm2 (Fig.3C-E, respectively), the number of well-formed tubes were substantially decreased to 10 ± 7 and 13 ± 3 tubes/field in S1- and S2-EC, respectively (Fig.3C). This characteristic was also projected into a marked reduction in the number of branch points as well as smaller tube areas when tube formation ability of SSc-EC was assessed (Fig.3D, E). These results suggested a defective angiogenic capacity for SSc-EC.

Downregulation of VE-cadherin and inability to form tubular network in SSc-derived ECs led us to examine VE-cadherin signaling and regulation.VE-cadherin/β-catenin signaling regulates the expression of matrix metalloproteinases (MMPs) in ECs during angiogenesis (17). The importance of MMPs in angiogenesis as well as possible angiogenesis-related changes in the expression of MMPs and VE-cadherin, was reported previously (18). In our *in vitro* differentiated SSc-ECs, while the expression of *MMP1* was 2.4-fold upregulated in S1- EC, *MMP9* expression showed 12-fold decrease compared to C1-EC, suggesting a dysregulation of *MMP1* and *MMP9* expression in S1-EC (Fig.3F). Scleroderma vessels have abnormal ECs which express regulator of G protein signaling 5 (RGS5), a protein associated with vascular rarefaction, but lack normal VE-cadherin expression (19). Interestingly, relative expression of *RGS5* was similar in C1-and S1-EC characterized in this study (Fig.3F). Moreover, the relative expression of *endothelin 1* (*EDN1*), which is involved in vascular remodeling of SSc (20), showed similar patternin S1- and C1-EC (Fig.3F).

Multiple mechanisms are involved in regulation of VE-cadherin including mammalian target of rapamycin (MTOR) and phosphoinositide-3 kinase (PI3K) signaling (21). In order to address the mechanism involved in the downregulation of VE-cadherin in S1-EC, the expression of PI3KCA (PI3K catalytic subunit alpha) and MTOR was assessed in iPSC-derived EC. Transcriptional analyses showed significant upregulation of *MTOR* and *PI3KCA* in S1-EC compared to C1-EC. Furthermore, the expression of *SNAI1*, a transcriptional repressor of VE-cadherin, was markedly increased in SSc-EC, indicating the possible role of *SNAI1* in downregulation of *VE-cadherin* (Fig.3F).

### Systemic sclerosis induced pluripotent stem cells could generate functional cardiomyocytes

**Fig.4:**
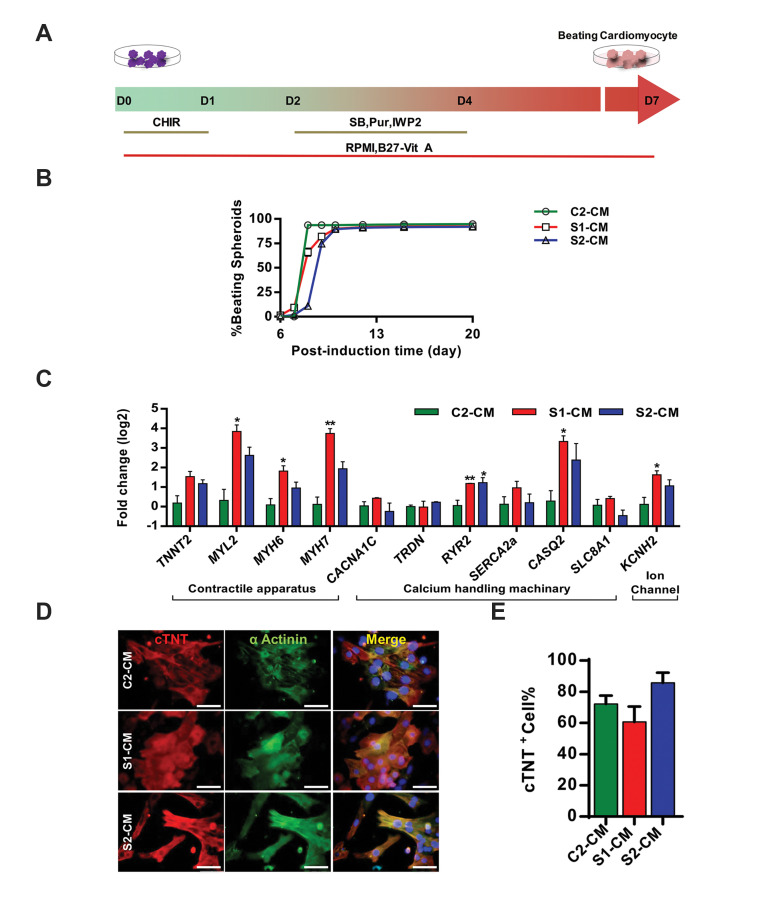
Generation and characterization of SSc iPSC-derived CM. **A. **A schematic diagram of the static suspension culture system used to induce cardiac differentiation of hiPSC lines. Five days after expansion in hESC medium, hiPSC aggregates (average size, 175 ± 25 μm) were transferred to low-attachment dishes containing differentiation medium and treated with 12 µM CHIR99021 for 1 day. After 1-day rest, the aggregates were treated with IWP2, SB431542, and purmorphamine (5 µM each) for 2 days, after which the media was exchanged every 2-3 days,** B.** The percentage of beating spheroids over the experimental period (n=3, biological replicate), **C. **Expression of cardiac markers in iPSC-derived CM on differentiation day 30 as measured by qRT-PCR. Fold change was calculated by ∆∆Ct method and expression of each gene was normalized against *GAPDH* (n=3, biological replicate), **D. **α-actinin (green) and troponin T (red) immunostaining of iPSC-derived CM on day 30 of cardiac differentiation. Nuclei were counterstained with DAPI (blue) (scale bar: 50 µm), and E. Flow cytometry analysis of cTnT^+^ cells showed same cardiac differentiation efficiency for SSc iPSC and control iPSC (n=3, biological replicate). All data are represented as mean ± SEM. Comparisons were made by one-way analysis of variance or unpaired t test when appropriate (* ; P<0.05 and * *; P<0.01 shows significant differences versus healthy control). SSc; Systemic sclerosis, iPSC; Induced pluripotent stem cells, CM; Cardiomyocytes, hiPSC; Human induced pluripotent stem cells, hESC; Human embryonic stem cells, qRT-PCR; Quantitative real-time polymerase chain reaction,C2-CM; Healthy control iPSC-CM, S1-CM; SSc1 iPS2-CM, and S2-CM; SSc2 iPS3- CM.

iPSC lines were subjected to cardiomyocyte differentiation in static suspension culture using a SM-based protocol (Fig.4A) which resulted in generation of spontaneously beating spheroids on day 7 that expanded to about 90% of spheroids on differentiation day 10. The ratio of beating spheroids was calculated daily and plotted for all three hiPSC lines (Fig.4B); this ratio peaked on day 10 (93.6 ± 1, 89.6 ± 0.9 and 90 ± 1.1% for healthy control iPSC-derived cardiomyocytes [C2-CM], S1-CM and S2-CM, respectively) showing their similar cardiac differentiation potency. The efficiency of cardiogenic differentiation was evaluated in C2 and SSc iPSC by assessing the gene and protein expression of cardiac specific markers (Fig.4C-E). All C2-iPSC, S1-iPS2 and S2-iPS3 derived CM expressed cardiac specific genes encoding cardiac structural proteins namely; *TNNT2* (cardiac type of troponin),* MYL2* (myosin light chain 2) and* MYH* (myosin heavy chain), encoding calcium handling proteins; *SERCA* (sarco/endoplasmic reticulum Ca^2+^-ATPase), *SLC8A1* (solut carrier family 8 of member A1), *CACNA1C* (calcium voltage-gated channel), *RYR2* (ryanodine receptor), *TRDN* (Triadin) and *CASQ* (calsequestrin), and encoding ion channels; *KCNH2* (potassium voltage-gated channel). While* MYL2, MYH6* and *MYH7* were upregulated in S1-CM, relative expression of these contractile apparatus-related genes were similar in S2-CM and C2-CM (Fig.4C). Furthermore, *MYH6/MYH7* ratio was decreased by 4- and 2-fold in S1-CM and S2-CM, respectively compared to C2-CM. While expression of *CASQ2* and *KCNH2* was substantially higher in S1-CM, *RYR2* expression was markedly elevated in both S1- and S2-CM. On the other hand, expression levels of other genes encoding calcium handling proteins (*CACNA1C, TRDN, SERCA,* and *SLC8A1*) were similar in all three hiPSC-derived CM. Protein expression of cTNT showed a similar pattern to that of its gene expression as reflected in immunostaining images; also, both SSc and C2 iPSC lines generated similar abundance of cTNT^+^ CM (Fig.4D, E). Altogether, these results indicated that SSc iPSC and healthy control iPSC have the same cardiogenic differentiation potential.

### Systemic sclerosis induced pluripotent stem cells-derived CM revealed normal excitation-contraction coupling

**Fig.5:**
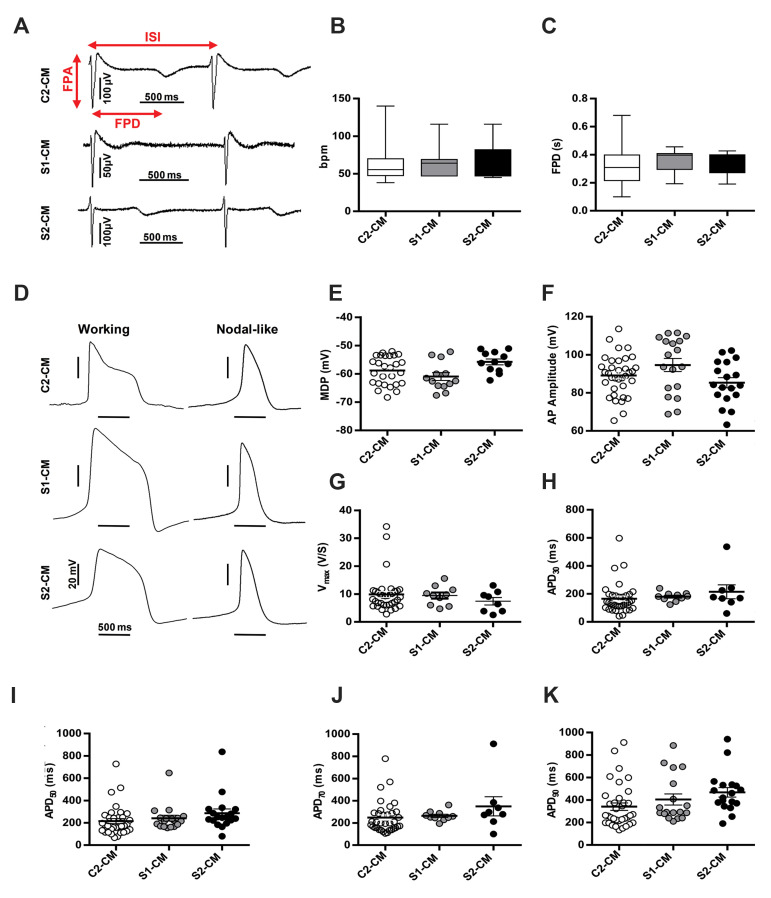
Electrophysiological properties of SSc iPSC-derived CM. **A.** Representative extracellular FP recorded from iPSC-derived beating spheroids using MEA at baseline, **B, C. **Electrophysiological features of cardiomyocytes as assessed by MEA revealed similarities in spontaneous beating rate (bpm) and FPD between SSc iPSC-derived CM and C2-CM, **D.** Representative AP recorded from single CM using whole-cell mode of patch-clamp technique, and **E-K.** Statistical analyses of action potential characteristics showed no significant differences between C2- and SSc iPSC-derived CM. All data are presented as mean ± SEM. Comparisons were made by one-way analysis of variance (ANOVA). SSc; Systemic sclerosis, iPSC; Induced pluripotent stem cells, CM; Cardiomyocytes, FP; Field potential, MEA; Multielectrode array, FPD; Field potential duration, AP; Action potential, ISI; Inter spike Interval, FPA; Field potential amplitude, MDP; Maximal diastolic potential, APA; Action potential amplitude, Vmax; Maximal upstroke velocity, APD _30-70_; Action potential duration measured at 30-90% of repolarization, C2-CM; Healthy control iPSC-CM, S1-CM; SSc1 iPS2-CM, and S2-CM; SSc2 iPS3- CM. (n≥7 in MEA experiments and n ≥ 18 in patch-clamp experiments).

To evaluate the functional properties of differentiated CM, two components of excitation-contraction coupling were studied. FPs generated by spontaneously beating spheroids were recorded to study the electrophysiological properties of SSc and C2-CM (Fig.5A). All differentiated CM derived from either SSc iPSC or control iPSC, showed a normal beating frequency of about 65 beats per minute (bpm) (Fig.5B). FP duration (FPD) which provides information on the repolarization phase of cardiomyocytes’ excitation and has been reported to be well correlated with AP duration obtained from a single cardiomyocyte (22), did not differ between SSc-and C2-derived cardiac spheroids (Fig.5C). Furthermore, single spontaneously beating CM were evaluated for their AP. Two types of AP, one specific for working CM and the other specific for nodal-like cells, were observed in C2-CM, S1-CM and S2-CM (Fig.5D). Various AP parameters such as maximal diastolic potential (MDP), upstroke velocity (V_max_) and AP duration at different time-points of repolarization (APD_x_) as well as APD_90_/ APD_50_ ratio were used for AP classification (23). Analysis of AP parameters showed that working CM were highly frequent in both C2- and SSc iPSC-derived CM (Fig.5E-K). Electrophysiological characteristics of iPSC-derived CM are summarized in Tables S5 and S6 (See Supplementary Online Information at www.celljournal.org).

Ca^2+^ transients as another component of excitation-contraction coupling was characterized in C2-CM, S1-CM and S2-CM. Ca^2+^ transient amplitude (Fig. S4A,B, See Supplementary Online Information at www.celljournal.org) and CTD80 did not significantly differ between patient-specific and healthy CM (Fig. S4C, See Supplementary Online Information at www. celljournal.org). Furthermore, fractional Ca^2+^ release which represents the ratio of active intracellular Ca^2+^ bulk to whole intracellular Ca^2+^, did not vary betweenC1-CM, S1-CM and S2-CM (Fig.S4D, See Supplementary Online Information at www.celljournal.org). Fractional Ca^2+^ release is basically defined as the ratio of the excitation-induced Ca^2+^ release to caffeine-induced Ca^2+^ release which depletes all intracellular Ca^2+^ stores. 

###  Systemic sclerosis induced pluripotent stem cells-derived CM were responsive to cardioactive drugs

**Fig.6:**
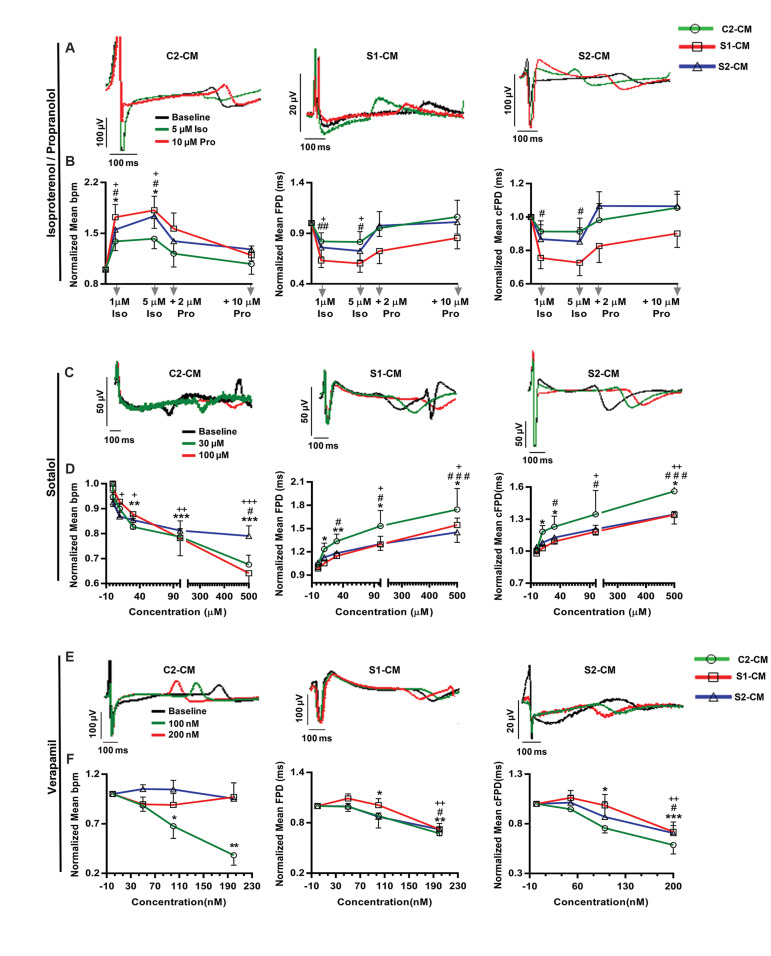
Response of SSc iPSC-derived CM to cardioactive drugs. **A.** Representative MEA traces showing FP shortenings induced by Iso treatment followed by FP prolongation after Pro administration, **B.** Iso significantly increased the beating frequency of CM derived from both SSc and control iPSC, but this increment was reversed by Pro. Iso reduced cFPD which was prolonged by Pro in hiPSC-derived CM, **C.** The prolongation of FP caused by sotalol, **D.** Reducing effect of sotalol on beating frequency. Treatment with sotalol prolonged the FPD and cFPD, **E.** The shortening of FP caused by verapamil, and F. Verapamil reduced beating rate of C2-CM and shortened the FPD and cFPD in iPSC-derived CM. All data are represented as mean ± SEM. Comparisons were made by one-way analysis of variance (ANOVA) or unpaired t test when appropriate (n ≥ 3, biological replicate). *; Shows significant differences of C2-CM, #; Significant differences of S1-CM and +; Significant differences of S2-CM following drug application (+, #, *; P<0.05, ##, ++, **; P<0.01, ###, +++, ***; P<0.001), SSc; Systemic sclerosis, iPSC; Induced pluripotent stem cells, MEA; Multielectrode array, FP; Field potential, CM; Cardiomycyte, C2-CM; Healthy control iPSC-CM, S1-CM; SSc1 iPS2-CM, S2-CM; SSc2 iPS3- CM, Iso; Isoproterenol, Pro; Propranolol, bpm; Beats per minute, FPD; Field potential duration, and cFPD; Corrected FPD (corrected according to the Bazett Formula).

Heart rate and contractility are regulated by the autonomic nervous system (22). To study the responsiveness of differentiated CM to neurohormonal regulation, the effects of a β-stimulant and a β-blocker agent (isoproterenol and propranolol, respectively) on C2-CM, S1-CM and S2-CM were assessed. Baseline extracellular FP was recorded for each spontaneously beating spheroid mounted on the electrodes of a MEA plate followed by recordings in the presence of serial concentrations of isoproterenol (Iso). The FP recording was performed 300 seconds after Iso application and antagonized by serial concentrations of propranolol (Pro). Iso and Pro concentrations were chosen based on previous studies (22). β_1_ -adrenergic stimulation by Iso substantially increased the spontaneous beating frequencies of iPSC-derived CM (5 µM Iso vs. baseline; 1.4-1.8, and 1.7 fold in C2- CM-S1, CM and S2-CM-respectively) which were antagonized by Pro as reflected by substantial reduction of beating rates to values approaching baseline frequencies. Furthermore, Iso administration caused significant shortening of FPD and cFPD which were reversed by Pro treatment (Fig.6A, B). Thus, SSc iPSC-derived CM responded to β-adrenergic agonist and antagonist in a similar manner to C2-CM. Also, the effect of sotalol as a cardioactive drug that blocks hERG channel was assessed on SSc iPSC-derived CM. Serial concentrations of sotalol (100 nM, 10 µM, 30 µM, 100 µM, and 500 µM) caused a gradual reduction of beating frequencies which accompanied by significant concentration-dependent FPD prolongation (Fig.6C, D). Verapamil as an L-type calcium channel blocker, was also added to CM differentiated from SSc iPSC. While serial concentrations of verapamil (50, 100, and 200 nM) induced a dose-dependent reduction of beating frequencies in C2-CM, it produced no effects on beating cycles of S1- and S2-CM. However, verapamil at concentrations ≥ 100 nM caused a significant reduction of FPD and cFPD in both patient-specific and control iPSC-derived CM (Fig.6E, F). Drug concentrations were chosen based on pervious works (24). Altogether, these results indicated that SSc iPSC-derived CM, are responsive to some important cardioactive drugs.

## Discussion

Here, we reported the generation of iPSC lines from two patients with SSc and their endothelial and cardiac differentiation. ECs were successfully derived from iPSC using a cocktail of growth factors and SMs that target signaling pathways involved in vascular system development, *in vivo*. Although SSc iPSC possessed the same pluripotency characteristics as their healthy counterparts, they showed different EC differentiation potentials. Furthermore, we found that angiogenic activity was significantly reduced in SSc-EC. Although there were no alterations at the primary stages of endothelial differentiation as reflected in similar expression of KDR, maturation phase of ECs was altered as presented in downregulation of VE-cadherin and loss of tube formation. Consistent with our results, Fleming et al. (25) stained endothelium and showed loss of VE-cadherin from some vessels of SSc patients. In contrast, Wang et al. (26) did not observe alterations in VE-cadherin protein expression in SSc iPSC-derived EC. Moreover study, Cipriani et al. (27) characterized CD31^+^ sorted EC isolated from SSc patients and did not report impaired VE-cadherin expression.

The important role of VE-cadherin in ECs’ tube formation is known so as anti-VE cadherin significantly decreased the number of tube structures in human umbilical vein ECs (HUVEC) (28). Importantly, VE-cadherin knockout resulted in defective EC maturation in animal models (29). Furthermore, Montero-Balaguer et al. (30) studied VE-cadherin function by making a knockdown of VE-cadherin in zebrafish using morpholino. They showed that partial VE-cadherin inactivation leads to vascular fragility, thus total amount of expressed protein is essential for vascular stability.

The role of MTOR in vascular development was previously reported (31). Bieri et al. (21) reported the regulatory effect of MTOR and PI3K signaling in VE-cadherin expression of HUVEC. Despite their report, we did not observe a similar pattern for PI3K, MTOR and VE-cadherin expression in SSc-EC suggesting there might be another mechanism involved in VE-cadherin regulation in SSc-ECs. It should be noted that in fibroblasts obtained from SSc patients, the expression of MTOR is elevated and it is involved in fibrotic response. Indeed, blockade of MTOR pathway is being studied as a potential therapeutic approach for scleroderma (32, 33).

Multiple mechanisms are involved in VE-cadherin regulation including Twist/Slug/Snail family which are transcriptional repressors of VE-cadherin gene (34). Lopez et al. (34) investigated the cause of VE-cadherin downregulation in ECs exposed to breast cancer cells-conditioned media and found direct repression of VE-cadherin promoter by Twist/Slug/Snail family. In the current study, we also observed upregulation of snail1 which might be one of the regulatory mechanisms under lying VE-cadherin downregulation in SSc-EC.

Absence of VE-cadherin expression and subsequent defective angiogenic activity of SSc-EC motivated us to assess the effect of VE-cadherin signaling on MMPs expression, which play multiple roles in angiogenesis (35). Kiran et al. (18) reported a reciprocal relationship between VE-cadherin and MMPs during angiogenesis. There are conflicting data on MMPs expression in scleroderma. While some researchers reported downregulation of MMP1 in scleroderma patients with increases in the levels of tissue inhibitor of MMP (TIMPs) (36), other studies reported the increased expression of MMP1 in SSc fibroblasts compared to healthy fibroblasts (37). Moreover, Kim et al. (38) investigated the expression of MMP9 in 42 SSc patients and observed elevated levels of MMP9. In contrast, Fuzii et al. (39) found decreased expression of MMP9 in dermal fibroblasts of SSc patients. In the present study, we also observed alterations in MMP1 and MMP9 expression in patients’ iPSC-derived EC; however, no reciprocal relation between MMP9 and VE-cadherin expression was found.

As majority of SSc patients suffer from cardiac involvements (6), we also investigated the cardiac differentiation of SSc iPSC and found similar cardiogenic potential when comparing these cells and the healthy control iPSC. A substantial percentage of differentiated CM was positive for the cardiac marker cTNT, in all three hiPSC-derived CM indicating the successful cardiac differentiation of SSc iPSC. The time course required for generation of beating CM from SSc iPSC and the efficiency of cardiogenesis were similar to those observed for healthy control. A similar pattern for cardiac specific markers and ion channels expression was observed in SSc iPSC-derived CM and C2-CM. Also, key components of the excitation-contraction complex were similarly expressed in patients and healthy differentiated CM. Notably, the expression level of MYH6, MYH7 and MYL2 was increased in S1-CM and the MYH6/MYH7 ratio was lower in both patient-derived CM compared to C2-CM which may indicate more mature phenotype of SSc iPSC-derived CM; however, the similar functional properties were found in all cardiomyocytes. SSc-derived CM also exhibited functional ion channels resulting in appropriate response to pharmacologically active compounds.

## Conclusion

The present study reports the successful differentiation of SSc iPSC into endothelial and functional cardiac cells that would provide a unique opportunity for mechanistic studies of scleroderma pathogenesis and possible targeted drug discovery.

## Supplementary PDF


