# Supplementary material for: A Novel Insight into Endothelial and Cardiac Cells Phenotype in Systemic Sclerosis Using Patient-Derived Induced Pluripotent Stem Cell
Source: Cell J. 2021 Jul 17;23(3):273–87. doi: 10.22074/cellj.2021.7244 (PMC8286459; doi:10.22074/cellj.2021.7244)
Supplement: Supplementary file 1 [file Cell-J-23-273-s01.pdf]

Supplementary Information for

A Novel Insight into Endothelial and Cardiac Cells Phenotype in Systemic Sclerosis Using Patient-Derived Induced Pluripotent Stem Cell

Sedigheh Gholami, Ph.D.[1, 2], Zahra Mazidi, M.Sc.[1], Sara Pahlavan, Ph.D.[1], Fariba Moslem, M.Sc.[1], Mahya Hosseini, M.Sc.[1], Adeleh Taei, Ph.D.[1], Mahdi Hesaraki, M.Sc.[1], Maryam Barekat, M.D.[3], Nasser Aghdami, M.D., Ph.D.[3]*, Hossein Baharvand, Ph.D.[1, 2]*

1. Department of Stem Cells and Developmental Biology, Cell Science Research Center, Royan Institute for Stem Cell Biology and Technology, ACECR, Tehran, Iran
2. Department of Developmental Biology, University of Science and Culture, Tehran, Iran
3. Department of Regenerative Medicine, Cell Science Research Center, Royan Institute for Stem Cell Biology and Technology, ACECR, Tehran, Iran

**Corresponding Addresses: P.O.Box: 16635-148, Department of Regenerative Medicine, Cell Science Research Center, Royan Institute for Stem Cell Biology and Technology, ACECR, Tehran, Iran*
P.O.Box: 16635-148, Department of Stem Cells and Developmental Biology, Cell Science Research Center, Royan Institute for Stem Cell Biology and Technology, ACECR, Tehran, Iran
Emails: nasser.aghdami@royaninstitute.org, baharvand@royaninstitute.org

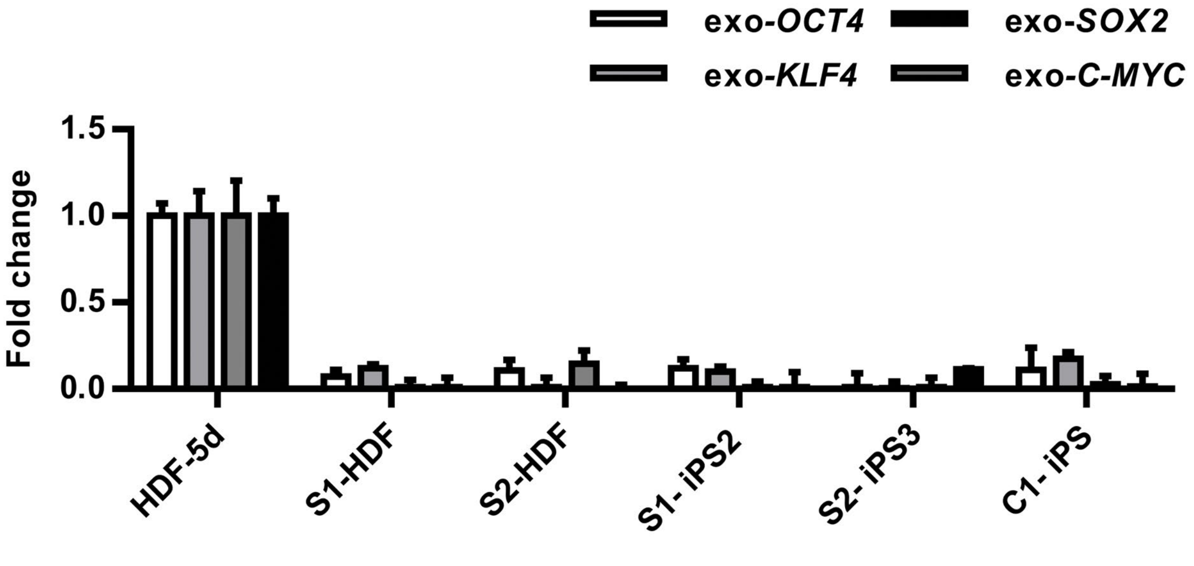

Fig.S1: Characterization of SSc iPSC. qRT-PCR analyses of retroviral transgene expression in freshly infected fibroblast (assessed five day post-infection; HDF 5d) and iPSC lines showed silencing of the exogenous genes in derived SSc iPSC in comparison to HDF 5d. Uninfected HDF (S1-HDF and S2-HDF) were used as negative controls. Data are presented as mean ± SEM, n=3, biological replicate. iPSC; Induced pluripotent stem cells, C1-iPS; Healthy control-iPS, S1-iPS2 (patient 1) and S2-iPS3 (patient 2) derived iPS. SSc; Systemic sclerosis, qRT-PCR; Quantitative real-time polymerase chain reaction, and HDF; Human dermal fibroblasts.

Table S1: SSc patients' clinical characteristics

**Characteristic**	**S1**	**S2**
Age (Y)	27	21
Sex (F/M)	F	F
Ethnic origin	Asian	Asian
Weight/BMI (kg)	43	44
MRSS	20	22
RSD	Light	high
Time since diagnosis (Y)	7	8
Type of SSc	Diffuse	Diffuse
CXR	ND	Normal
ANA	+	+
Major organ involvement:		
Lung		
Dyspnea	Activity	Activity
Forced vital capacity (%) predicted)	ND	62
DLCO (% predicted)	ND	80
Crackle	ND	+
Kidney		
Protein	+	-
Blood creatinine	0/6	0/8
Heart		
Pericardial effusion	-	-
LVEF	50-55%	55%
PAP (mm Hg)	45	26
Cardiac symptoms (Palpitation, chest pain)	-	ND
Cardiac risk factors:		
DM	-	-
HLP	-	-
History of HTN	-	-
HTN	-	-
Blood pressure (mmHg)	85/60	95/60
NYHA (I, II, III, VI)	II	II

BMI; Body mass index, MRSS; Modified rodnan skin score, RSD; Raynauds condition score, SSc; Systemic sclerosis, CXR; Chest-X ray, ANA; Anti-nuclear antibodies, LVEF; Left ventricular ejection fraction, PAP; Pulmonary arterial pressure, DM; Diabetes mellitus, HTN; Hypertension, HLP; Hyperlipidemia, NYHA; New York Heart Association, DLCO; Diffusing capacity of the lung for carbon monoxide, and ND; Not determined.

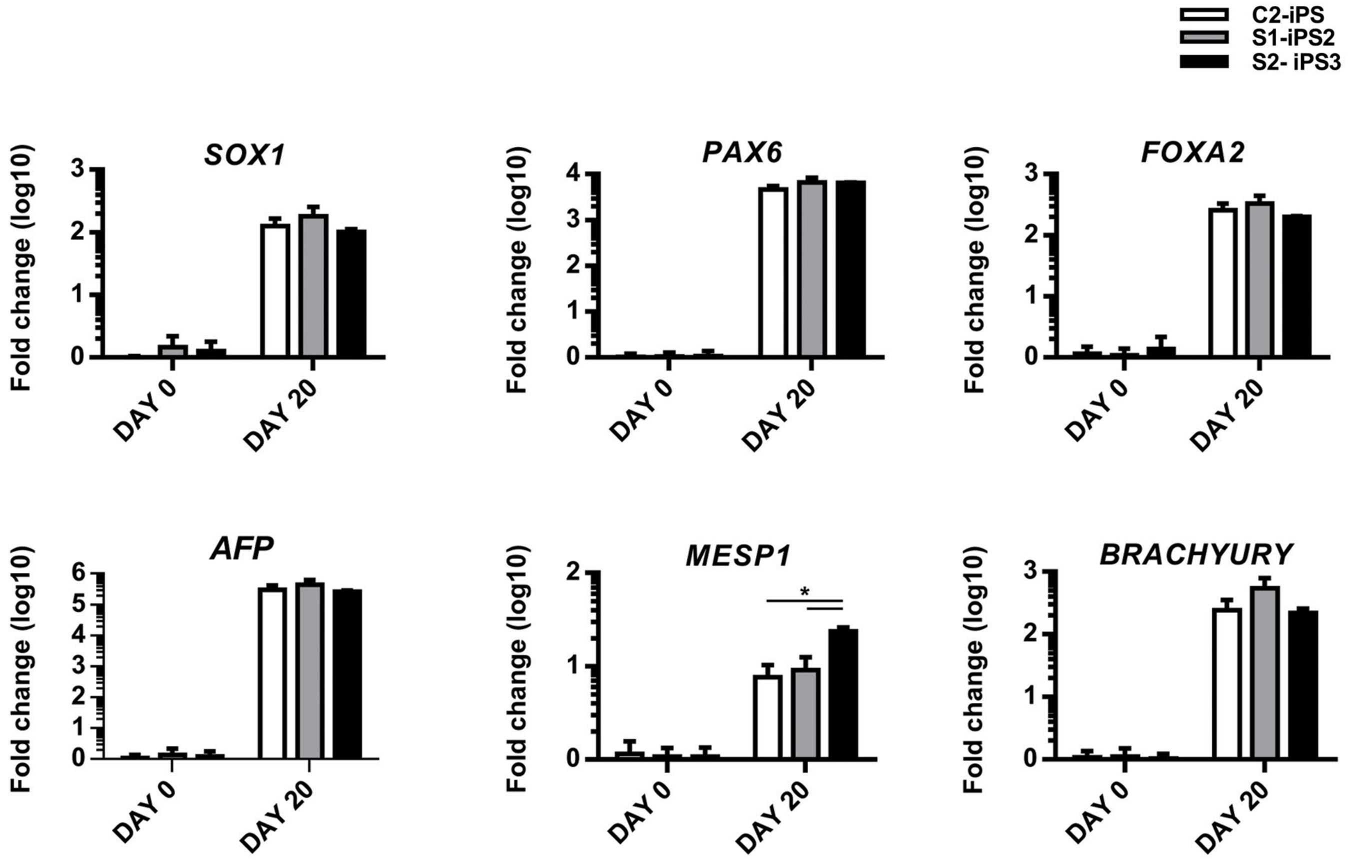

Fig.S2: Spontaneous differentiation of SSc iPSC by EB formation. Relative expression levels of ectoderm (*SOX1* and *PAX6*), endoderm (*FOXA2* and *AFP*) and mesoderm (*MESP* and *Brachyury*) genes in the iPSC lines as measured by qRT-PCR. Fold change was calculated by ΔΔCt method and expression of each gene was normalized against *GAPDH*. Data are represented as mean ± SEM. Comparisons were made by one-way analysis of variance (ANOVA) (*; P<0.05, n=3, biological replicate). iPSC; Induced pluripotent stem cells, C2-iPS; Healthy control-iPS, S1-iPS2 (patient 1) and S2-iPS3 (patient 2) derived iPS. SSc; Systemic sclerosis and qRT-PCR; Quantitative real-time polymerase chain reaction.

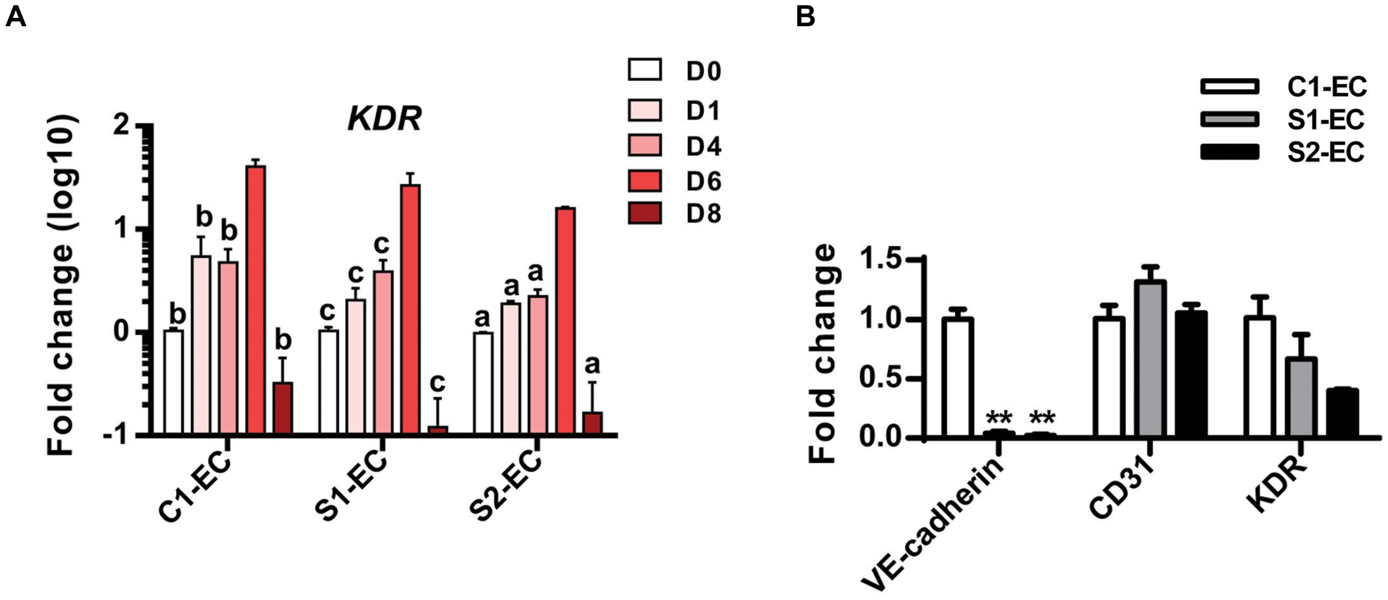

Fig.S3: Successful differentiation of SSc iPSC to endothelial lineage. **A.** qRT-PCR analysis showed the peak of *KDR* expression on differentiation day 6 (D6). Comparisons were made by two-way analysis of variance (ANOVA) (a; P<0.001, b; P<0.01, and c; P<0.05 show significant differences vs. D6) and **B.** Expression of endothelial markers in SSc-EC (KDR on d6, CD31 and VE-cadherin on d8 of endothelial differentiation). Data are represented as mean ± SEM. Comparisons were made by one-way analysis of variance (ANOVA) (**; P<0.01, n=2, biological replicate). iPSC; Induced pluripotent stem cells, EC; Endothelial cells, C1-EC; Healthy control iPSC-EC, S1-EC; SSc1 iPS2-EC, S2-EC; SSc2 iPS3- EC, SSc; Systemic sclerosis, and qRT-PCR; Quantitative real-time polymerase chain reaction.

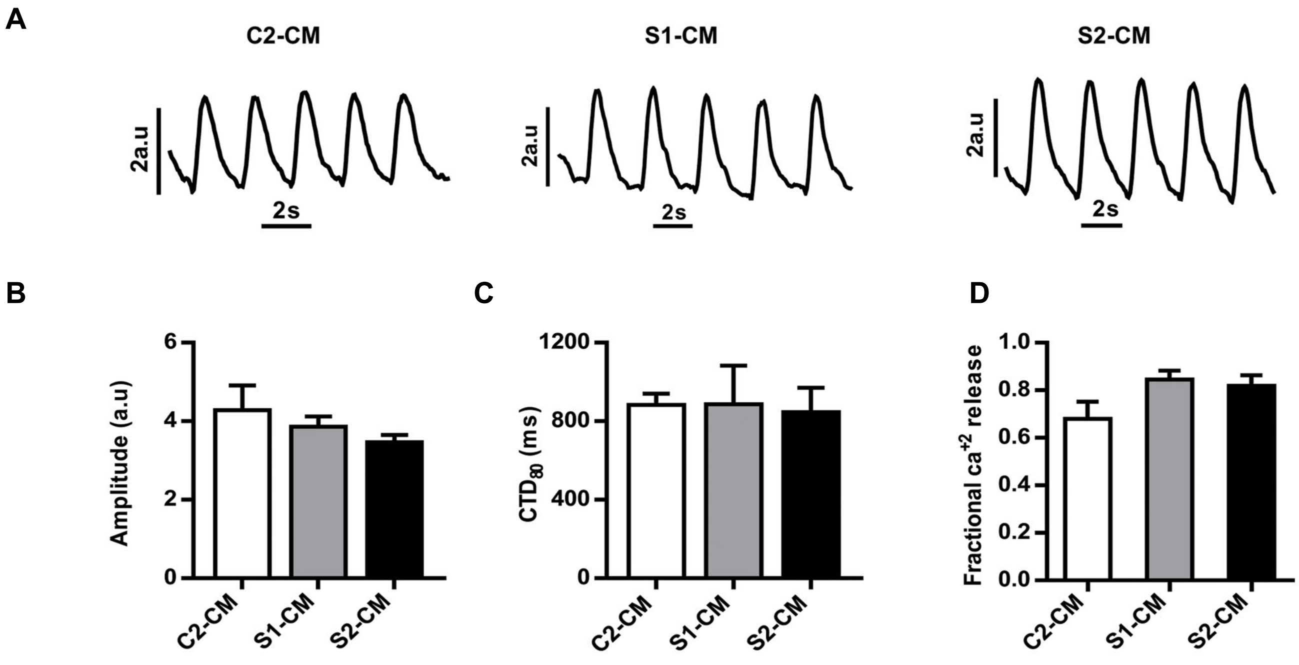

Fig.S4. Functional characterization of SSc iPSC-derived CM. **A.** Representative traces of Ca^{2+} transients recorded in iPSC-derived CM **B.** Ca^{2+} transient amplitude **C.** CTD80 (Ca^{2+} transient duration at 80% decay) and **D.** Fractional Ca^{2+} release of SSc-CM were similar to C2-CM. Data are represented as mean ± SEM. Comparisons were made by one-way analysis of variance (ANOVA), n≥3. CM; Cardiomyocytes, C2-CM; Healthy control iPSC-CM, S1-CM; SSc1 iPS2-CM, and S2-CM; SSc2 iPS3- CM, CTD80; calcium transient duration at 80% decay. SSc; Systemic sclerosis and iPSC; Induced pluripotent stem cells.

Table S2: List of antibodies used for immunofluorescence staining and flow cytometry analyses

Antibody	Company	Cat. number
TRA-1-60	Chemicon	MAB4360
TRA-1-81	Chemicon	MAB4381
Oct-4	Santa Cruz Biotechnology	SC-5279
Nanog	Santa Cruz Biotechnology	SC-30331
vWF	Beckton Dickenson (BD)	555849
CD144 (VE-cadherin)	Beckton Dickenson (BD)	560411
CD31	Abcam	Ab 28364
CD31	Beckton Dickenson (BD)	555444
CD144(VE-cadherin)	Beckton Dickenson (BD)	555661
VEGFR2	R&D	FAB357P
cTNT	Abcam	ab64623
α-actinin	Sigma-Aldrich	a7811
anti-mouse IgM	Sigma	F9259
Anti-goat	Invitrogen	A11055
Anti-rabbit	Santa Cruz Biotechnology	SC2780
anti-mouse IgG	Sigma	F9006
anti-mouse IgG	Invitrogen	A11004
Rat anti-mouse IgG1-FITC	Beckton Dickenson (BD)	04611
Donkey anti-mouse Alexa 488	Invitrogen	A21202
Donkey anti-Goat Alexa 568	Invitrogen	A11057
Donkey anti-Goat Alexa 546	Invitrogen	A11056

Table S3: List of primers used for gene expression assay

Gene symbol	Forward (Sequences 5´- 3´)	Reverse (Sequences 5´- 3´)
TNNT2	ATGATGCATTTTGGGGGTTA	CAGCACCTTCCTCCTCTCAG
MYH7	ACC CAA GTT CGA CAA AAT CG	TAA GGG TTG ACG GTG ACA CA
MYH6	ATTGCTGAAACCGAGAATGG	CGCTCCTTGAGGTTGAAAAG
MYL2	CTTGGGCGAGTGAACGT	CTGGTCAACCTCCTCCTTG
SERCA2a	CATCAAGCACACTGATCCCGT	CCACTCCCATAGCTTTCCCAG
RYR2	GGCAGCCCAAGGGTATCTC	ACACAGCGCCACCTTCATAAT
SLC8A1	TCATAGCTGATCGGTTCATGTCC	CAGTTGTCTTGGTGGTCTCTC
KCNH2	CAACCTGGGCGACCAGATAG	GGTGTTGGGAGAGACGTTGC
CASQ2	CATTGCCATCCCCAACAAACC	AGAGTGGGTCTTTGGTGTTCC
TRDN	TCACAGAAGACATAGTGACGACG	TGGCAATAGAGCTTGCTGAAA
CACNA1C	AATCGCCTATGGACTCCTCTT	GCGCCTTCACATCAAATCCG
GAPDH	CTCATTTCCTGGTATGACAACGA	CTTCCTCTTGTGCTCTTGCT
SOX1	GTGTACCCTGGAGTTTCTG	TAGTCTGTGCCTCTAAAGTG
PAX6	GTC CAT CTT TGC TTG GGA AA	TAG CCA GGT TGC GAA GAA CT
MESP1	ACCTTCGAAGTGGTTCCTTG	TCCTGCTTGCCTCAAAGTGT
BRACHYURY	AATTGGTCCAGCCTTGGAAT	CGTTGCTCACAQACCACA
FOXA2 B	ATGCACTCGGCTTCCAGTAT	TGTTGCTCACGGAGCAGTAG
AFP	GCAGCCAAAGTGAAGAGGGAAGA	GTCATAGCGAGCAGCCCAAAGAAG
NANOG	CAGCTACAAACAGGTGAAGAC	TGGTGGTAGGAAGAGTAAAGG
OCT4	GTT CTT CAT TCA CTA AGG AAG G	CAA GAGCATCATTGA ACT TCAC
CD31	AGCAGTACCACTTCTGAACTCC	AGGAATTGCTGTGTTCTGTGG
KDR	AAGTATGTGACCCCAAATTCC	AGAACAACACTTGAAAATCTG
VE-cadherin	CTCCAACTCCATACTCCACTC	AGTCTCAAAGCAAGGTCTCAG
OCT4	CTGGGTTGATCCTCGGACCT	CACAGAACTCATACGGCGGG
NANOG	AAAGAATCTTCACCTATGCC	GAAGGAAGAGGAGAGACAGT
C-MYC	GCGTCCTGGGAAGGGAGATCCGGAGC	TTGAGGGGCATCGTCGCGGGA GGCTG
SOX2	GGG AAATGGAAG GGG TGCAAA GAGG	TTGCGTGAGTGTGGATGG GATTGGTG
KLF4	ACGATCGTGGCCCCG GAAAAGGACC	TGATTGTAGTGCTTTCTGGCTGGGCTCC
MTOR	TACAGGCACACATTTGAAGAAGCAG	TCTTCTCTCAGACGCTCTCCC
PI3KCA	CTCGAGTTAAACAGCATGCATTGAACTGAAAAG	GCGGCCGCCATCACTTTTTCCTTCTCCATCATTTC
EDN1	CAGCGTCCTCGTTCAAAACATT	CCCCAGATGAAAGAAGAGACCA
RGS5	AGCCAAGACCCAGAAAACCT	TTTGCCTTCTCAGCCATCTT
MMP1	AAAATTACACGCCAGATTTGCC	GGTGTGACATTACTCCAGAGTTG
MMP9	TGTACCGCTATGGTTACACTCG	GGCAGGGACAGTTGCTTCT
SNAI1	CCAGAGTTTACCTTCCAGCA	GATGAGCATTGGCAGCGA

Table S4: List of drugs used in pharmacological studies

Drug	Company	CAS. number
Isoproterenol hydrochloride	Sigma-Aldrich	51-30-9
Propranolol hydrochloride	Sigma-Aldrich	318-98-9
Sotalol hydrochloride	Sigma-Aldrich	959-24-0
Verapamil hydrochloride	Tocris	152-11-4
Caffeine	Sigma-Aldrich	58-08-2

Table S5: Baseline electrophysiology in spontaneously beating colonies assessed by MEA

Cell type	FPD, ms	bpm
C2-CM	338.4 ± 55	64.79 ± 7.58
S1-CM	353 ± 33	65.29 ± 9.15
S2-CM	326.3 ± 19	65.71 ± 10.03

Data are presented as mean ± SEM. MEA; Multielectrode array, iPSC; Induced pluripotent stem cells, bpm; Beats per minute, FPD; Field potential duration, CM; Cardiomyocytes, C2-CM; Healthy control iPSC-CM, S1-CM; SSc1 iPS2-CM, S2-CM; SSc2 iPS3- CM, C2-CM (n=14), S1-CM (n=7) and S2-CM (n=15).

Table S6: Baseline electrophysiology in single cardiomyocytes assessed by patch-clamp technique

Cell type	MDP, mV	APA, mV	APD_{30}, ms	APD_{50}, ms	APD_{70}, ms	APD_{90}, ms	Vmax, V/S
C2-CM	-58.84 ± 0.95	89.12 ± 1.79	165.7 ± 18.43	215.4 ± 22.57	248.1 ± 24.23	341.1 ± 32.74	9.858 ± 1.10
S1-CM	-60.96 ± 1.38	94.64 ± 3.46	180.1 ± 10.22	241.4 ± 26.32	264.3 ± 14.24	404.9 ± 49.00	9.450 ± 1.08
S2-CM	-55.78 ± 1.04	85.33 ± 2.63	214.7 ± 49.90	286.1 ± 37.82	349.7 ± 85.81	468.3 ± 42.83	7.413 ± 1.34

Data are presented as mean ± SEM. APA; Action potential amplitude, $APD_{30\text{-}90}$; Action potential duration at 30-90% of repolarization, MDP; Maximal diastolic potential, Vmax; Maximal upstroke velocity, CM; Cardiomyocytes, C2-CM; Healthy control iPSC-CM, S1-CM; SSc1 iPS2-CM, S2-CM; SSc2 iPS3-CM, C2-CM (n=35), S1-CM (n=18) and S2-CM (n=19).
